# Supplementary material for: INSIDER: Interpretable sparse matrix decomposition for RNA expression data analysis
Source: PLoS Genet. 2024 Mar 14;20(3):e1011189. doi: 10.1371/journal.pgen.1011189 (PMC10965063; doi:10.1371/journal.pgen.1011189)
Supplement: S1 Text — It provides technical details on enrichment analysis, gene selection in comparing expression differences between dementia and control across brain regions, and metagene selection for discovering heterogeneous effects of dementia on brain structures. Regarding the model selection of INSIDER, it demonstrates the effect of different numbers of iterations (e.g., 30, 50) on model selection. It also shows the capability of INSIDER to handle ordinal variables via simulation studies. Regarding the difference between INSIDER and PCA/factor analysis or GLM-based approaches, it presents an in-depth discussion on their differences. To demonstrate its wide applicability, results from analyzing gender differences in gene expression of the human brain with GTEx using INSIDER are provided. Further, derivations for random coordinate descent for elastic net problems and the RCD algorithm (Algorithm 1) for the elastic net with screening rules are also presented. (DOCX) [file pgen.1011189.s001.docx]

Supplementary Content

1. Technical details on enrichment analysis

Here we focus on the biological processes (BPs) enriched by genes with large effects. Specifically, for a given metagene $V_{i}$, the $i$-th row of $V$, we examine the biological processes (BPs) enriched by genes with large effects, that is, the genes with large absolute values for $V_{i}$, since these genes encode primary biological functions of the metagene. Therefore, enrichment analyses were conducted on the set of genes in the upper or lower 2.5% quantile of $V_{i}$ to explore up- or down-regulated BPs encoded by the metagene, respectively. Similarly, we used the above strategy to reveal the up- or down-regulated BPs encoded by adjusted expression profiles or differences in expression profiles. In the implementation, the function *enrichGO* from the R package *clusterProfiler* (v4.4.4) was utilized, with the “*ont*” parameter set to "BP".

1. Gene selection in comparing expression differences between dementia and control across brain regions

To examine whether gene expression across different brain regions is similarly affected by dementia. To answer this question, we first selected three genes (*NRGN*, *CAMK2A*, and *SHISA6*), which were selected by the below procedure. Specifically, the genes (~82 genes) enriched for the top five BPs (Fig 3B from the main text) were extracted, and then conducted t-tests to check the significance of differences in expression across different brain regions for each gene and then employed Simes’ test to obtain aggregated p-values for each gene, with the consideration of multiple testing. Thus, based on aggregated *p*-values significant at ~0.05, the three genes were selected.

We plotted the expression levels of the three genes between dementia and control across brain regions (S4 Fig). Surprisingly, the original expression levels of *CAMK2A* and *SHISA6* in the left HPC were significantly lower in dementia than in controls, but a similar phenomenon was not observed in the right part (S4 Fig). In short, our analysis suggests that gene expression of the left and right HPC may be affected differently by dementia.

1. Metagene selection for discovering heterogeneous effects of dementia on brain structures

In practice, we used the following strategy to select metagene selection for the study purpose. First, we obtained the profiles of the left HPC $E$ for dementia and control by multiplying the submatrix $W_{k}$ of interaction representation corresponding to the left HPC by gene representation $G$. In the above multiplication, only top-*N* metagene with the greatest difference in $W_{k}$ were used. The number of genes (*N*) is determined by the significance of p-values in the following-up enrichment analysis. We selected the number of top genes which leads to the most significant *p*-values. Here, $E$ is a matrix of two rows corresponding to the adjusted expression profiles of the left HPC for dementia and control. Then, we calculated the difference between the two rows of $E$ by $E_{1}-E_{2}$ and examined the down-regulated BPs enriched by the difference. For the right HPC, we follow the same procedure above to ensure consistency.

1. Exploring the effect of different numbers of iterations (e.g., 30, 50) on model selection

For demonstration with the BrainSpan dataset, we examined the effect of different numbers of iterations (e.g., 30, 50) on model selection and showed RMSE on the trainset and testset with 30 and 50 iterations for the rank $K$ chosen from 11 to 50 with step size 2. The results are presented in Figure 1 and Table 1. From the table and figure, we see that 30 iterations are sufficient to give consistent selection results, compared with more iterations (50 iterations).

Figure 1. RMSE on trainset and testset for different $K$ after 30 and 50 iterations in application to the BrainSpan data


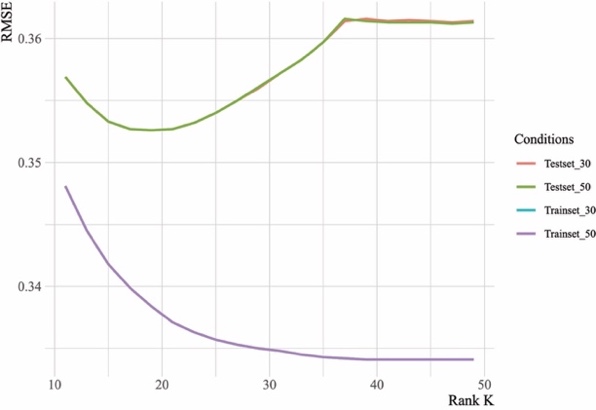


Table 1. RMSE on the trainset and testset for different choices of $K$ after 30 and 50 iterations in application to the BrainSpan data

| Rank *K* | Trainset  (30 iterations) | | Testset  (30 iterations) | Trainset  (50 iterations) | Testset  (50 iterations) |
| --- | --- | --- | --- | --- | --- |
| 3 | 0.40209 | 0.40450 | | 0.40209 | 0.40450 |
| 5 | 0.37800 | 0.38169 | | 0.37800 | 0.38169 |
| 7 | 0.36429 | 0.36994 | | 0.36429 | 0.36994 |
| 9 | 0.35457 | 0.36200 | | 0.35457 | 0.36200 |
| 11 | 0.34810 | 0.35686 | | 0.34810 | 0.35686 |
| 13 | 0.34450 | 0.35479 | | 0.34450 | 0.35479 |
| 15 | 0.34182 | 0.35330 | | 0.34182 | 0.35330 |
| 17 | 0.33992 | 0.35273 | | 0.33992 | 0.35273 |
| 19 | 0.33842 | 0.35261 | | 0.33838 | 0.35256 |
| 21 | 0.33713 | 0.35274 | | 0.33713 | 0.35274 |
| 23 | 0.33628 | 0.35320 | | 0.33628 | 0.35321 |
| 25 | 0.33571 | 0.35397 | | 0.33571 | 0.35397 |
| 27 | 0.33534 | 0.35498 | | 0.33534 | 0.35498 |
| 29 | 0.33503 | 0.35604 | | 0.33503 | 0.35609 |
| 31 | 0.33477 | 0.35723 | | 0.33477 | 0.35725 |
| 33 | 0.33454 | 0.35829 | | 0.33454 | 0.35830 |
| 35 | 0.33435 | 0.35968 | | 0.33434 | 0.35971 |
| 37 | 0.33417 | 0.36135 | | 0.33417 | 0.36159 |
| 39 | 0.33412 | 0.36155 | | 0.33412 | 0.36138 |
| 41 | 0.33412 | 0.36140 | | 0.33412 | 0.36128 |
| 43 | 0.33412 | 0.36149 | | 0.33412 | 0.36131 |
| 45 | 0.33412 | 0.36144 | | 0.33412 | 0.36130 |
| 47 | 0.33412 | 0.36134 | | 0.33412 | 0.36121 |
| 49 | 0.33412 | 0.36144 | | 0.33412 | 0.36127 |

1. The difference between INSIDER and PCA/factor analysis or GLM-based approaches

Here we would like to demonstrate the difference between INSIDER and PCA, as well as GLM-based approaches in depth. For illustration, assume that $Z^{N\times M}$ is the expression of $M$ genes for $N$ samples and that $X^{N\times p}$ is a covariate matrix for $p$ categorical variables, such as donor, tissue, phenotype.

In PCA, the variation of $Z^{N\times M}$ is modeled by ${Z^{N\times M}=H}^{N\times K}V^{K\times M}+\Theta$, where $\Theta$ is the matrix for noise. Both $H^{N\times K}$ and $V^{K\times M}$ are unobserved. After performing PCA/factor analysis, we have latent embeddings $H^{N\times K}$ for $N$ samples. For the GLM-based approaches for differentially expressed analysis, the variation of $Z^{N\times M}$ is modeled by $X^{N\times p}\beta^{p\times M}$. Here $\beta^{p\times M}$ is the coefficient matrix of $p$ covariates for $M$ genes and is used for differentially expressed analysis for the categorical covariates like phenotypes. For INSIDER, the variation of $Z^{N\times M}$ is approximated by $Z^{N\times M} \approx\left( X_{D}D+X_{P}P+X_{T}T+X_{W}W \right)V$. Here $X_{D},X_{P},X_{T},X_{W}$ are the dummy indicator matrices for donors, tissues, phenotypes, and the interaction between phenotypes and tissues, rows of $V$ are metagenes, which capture genes that co-express or work together to induce biological functions, and $D,P,T,W$ are the levels of expression of metagenes (i.e., sets of biological pathways) for different covariates. INSIDER also considers the sparsity and grouping effects of genes by introducing elastic net penalty.

We see that INSIDER separately models the variation from different known sources (e.g., donors, tissues, phenotypes, and their interactions) to capture the variation of $Z^{N\times M}$, represented by the terms $X_{D}DV$, $X_{P}PV$, $X_{T}TV$, and $X_{W}WV$. However, PCA/factor analysis ignores the source of variation in $Z^{N\times M}$, as it computes $H^{N\times K}$ for samples without further separating the source of variation with respect to known covariates. Thus, a direct comparison between INSIDER and PCA/factor analysis in real data analysis is not applicable. The matrices $D,P,T,W$ in INSIDER reveal the effect of the covariates on the metagenes (i.e., biological pathways), which PCA/factor analysis cannot provide.

As mentioned previously, GLM-based approaches are usually used for differentially expressed analysis in RNA-Seq studies. They compute the coefficient matrix $\beta^{p\times M}$ for $p$ covariates across $M$ genes. That is, $\beta$ measures the relationship between covariates and genes. However, INSIDER is a statistical approach for exploring the latent structure underlying $Z^{N\times M}$ with respect to known sources of variation. In INSIDER, the variation in $Z$ is modeled by $\left( X_{D}D+X_{P}P+X_{T}T+X_{W}W \right)V$. Here $X_{D},X_{P},X_{T},X_{W}$ are covariates. Rows of $V$ are metagenes and capture genes that co-express or work together to induce biological functions, so each row of $V$ corresponds a set of biological pathways. $D,P,T,W$ are the levels of expression of metagenes (i.e., sets of pathways) for different covariates. Therefore, INSIDER explores the effects of covariates on these metagenes (i.e., pathways) encoded by $V$. We see that INSIDER and GLM-based methods explore different levels of biological information in RNA-seq data. Therefore, a direct comparison between INSIDER and GLM-based methods in real data is also inapplicable.

1. Simulation studies on the capability of INSIDER in handling ordinal variables

We carried out simulation studies to show INSIDER can recover the monotonic trend for ordinal variables. In our simulation setup, we consider a gene expression matrix $Z$ (250×200), and variation in the matrix comes from two sets of covariates ($X_{E},X_{F}$), which are dummy matrices for two categorical variables, their interaction, and random noise. Their relationship is described by $Z=T+\Theta$, where $T=\left( X_{E}E+X_{F}F+X_{R}R \right)V$. The rank of latent space is set to 5, $E$ and $F$ are matrices of dimensions 50×5 and 5×5, respectively, and the dimension of $R$ for the interaction between the two sets of covariates is 250×5. $V$ is a 5×250 matrix for gene latent representations.

Here $X_{F}$ contains an ordinal variable with 5 levels, and the $i$-th row of $F^{5\times5}$ is generated from $N\left( i, 0.25 \right),\forall i=1,\ldots,5$, so $F$ is monotonic increasing across the 5 levels. All other matrices were generated from the standard normal distribution. The design matrices for $\{E, F,R\}$ are $X_{E}^{250\times50}$, $X_{F}^{250\times5}$, and $X_{R}^{250\times250}$, respectively. The noise matrix $\Theta$ (250×200) was generated from a normal distribution $N(0,\delta)$, and $\delta$ is the variance of the distribution. We considered $\delta$ equal to 0.1, 0.2, 0.3, 0.4, and 0.5 in our simulations.

The result of the above simulation is presented in the table below. From the table, INSIDER well recovered the monotonic trend of the ordinal variable $F$.

Table 2. The true and estimated mean for the 5 levels of the ordinal variable $F$ over the 5 latent variables

| Noise level | Ordinal variable $F$ | Level 1 | Level 2 | Level 3 | Level 4 | Level 5 |
| --- | --- | --- | --- | --- | --- | --- |
| 0 | True mean of row of $F$ | 0.9541 | 1.9988 | 3.0409 | 3.9389 | 5.0232 |
| 0.1 | Row mean of estimated $\hat{F}$ by INSIDER | 0.7350 | 1.7280 | 2.9561 | 3.7810 | 4.9520 |
| 0.2 |  | 0.7637 | 1.8268 | 3.0020 | 3.8456 | 4.9192 |
| 0.3 |  | 0.7567 | 1.8618 | 2.9883 | 3.8964 | 4.9510 |
| 0.4 |  | 0.7653 | 1.8421 | 3.0437 | 3.8846 | 4.9799 |
| 0.5 |  | 0.7538 | 1.8587 | 3.0131 | 3.9384 | 5.0150 |

In summary, it is a feasible solution for INSIDER to consider ordinal variables as unordered categorical variables.

1. Analysis of gender differences in gene expression of human brain with GTEx

In this application, we seek to explore gender differences in gene expression across 13 different brain regions from the GTEx data [1]. Details of data processing are shown in SC. The dimension of our processed data is 988×49,999 for samples from 318 different donors. For each brain region, it has 38 RNA-seq samples from either men or women. We modeled three covariates: brain region, gender, and gene. The number of ranks K of latent space selected by hyperparameter tuning is 12.

### *Metagenes reveal gender differences in gene expression of human brain*

To reveal gender differences in gene expression of human brain, we calculated the adjusted gene expression profiles for genders by multiplying gender representation with gene representation and then examined up- (Figure 2A) and down-regulated (Figure 2B) BPs enriched by the difference in gene expression between the male and the female.

On the one hand, expression levels of genes enriched for synapse activities and neurotransmitters are higher in men than in women across the brain regions included (Figure 2A). On the other hand, women have higher expression levels of genes contributing to immune responses, such as (defense) response to viruses and bacterium and humoral immune response, than men (Figure 2B).

Figure 2 Downstream analyses with results from the application to the GTEx data


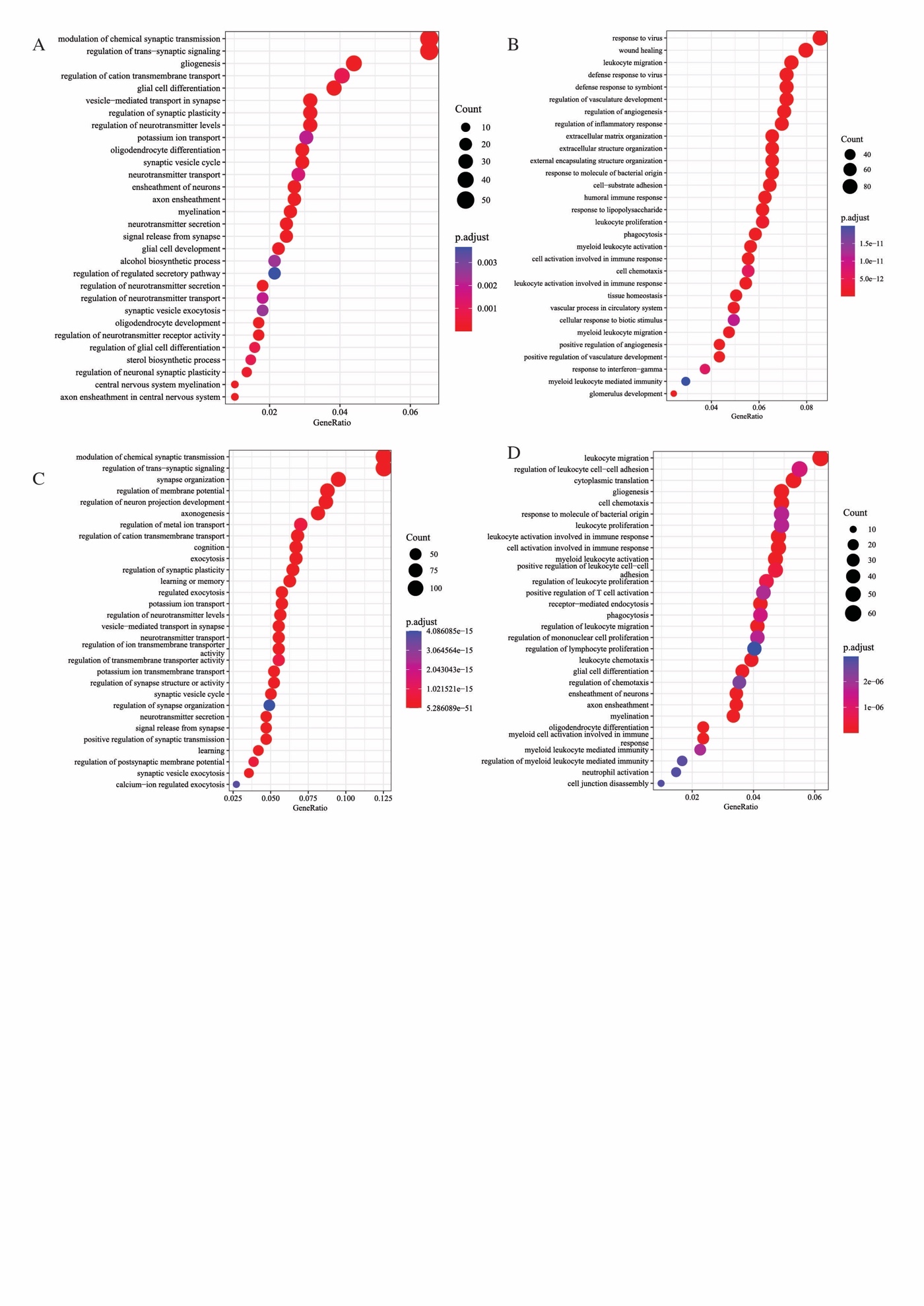


1. Figures A and B show the top 30 up- and down-regulated BPs enriched by the difference in gene expression between genders, respectively.
2. Figures C and D display the top 30 up- and down-regulated BPs enriched for the difference in gene expression of the frontal cortex (BA9) between genders, respectively.

### *Metagenes characterize functions of brain regions*

Previously, we revealed gender differences in gene expression of human brain utilizing metagenes. Additionally, metagenes also enable us to uncover the functions of brain regions included. Intuitively, if the loading of a specific metagene for one brain region is significantly greater than that of other brain regions, then the metagene can be utilized to characterize the brain region. Following the intuition, we highlighted several metagenes to demonstrate how to achieve this goal in practice.

First, we found that the loading of the 3^rd^ metagene is highly positive and much greater in the three subcortical nuclei (putamen, nucleus accumbens, and caudate) than in other brain regions. The top 30 BPs enriched by up-regulated genes of the metagene mainly fall into the following aspects: muscle contraction and system process, blood circulation and circulatory system, response to monoamine, catecholamine, and dopamine, synaptic transmission and signaling, and neurotransmitter transport (Figure 3A), highlighting their roles in human motor and emotions.

Meanwhile, we also examined the top 30 up-regulated BPs enriched by the 4^th^ (Figure 3B) and 6^th^ (Figure 3C) metagenes since the loadings of the 4^th^ metagene for the hypothalamus and 6^th^ metagene for HPC are highly positive and much greater than for other brain regions. The up-regulated BPs enriched by the 4^th^ metagene primarily involve hormone secretion and transport, calcium ion homeostasis, and catecholamine and monoamine transport (Figure 3B), which is consistent with the role of the hypothalamus in homeostasis [2]. Meanwhile, the up-regulated BPs enriched by the 6^th^ metagene involve learning and memory, synaptic transmission and signaling, and dendrite development (Figure 3C), revealing the key functions of HPC.

Moreover, we also explored the top 30 up-regulated BPs enriched by the 12^th^ metagene, whose loading is highly negative in the spinal cord. The up-regulated BPs involve the following biological activities: learning, memory, cognition, synaptic transmission and signaling, and neurotransmitter transport and secretion (Figure 3D), implying a much lower involvement of the spinal cord in human cognitive function, compared with other brain regions.

### *Explore gender differences in the expression of specific brain regions*

In the previous subsection, we revealed gender differences in gene expression of human brain across all brain regions included. Still, psychiatrists might be more interested in the gender difference in a specific brain region since genders show differences in mental disorder prevalence [3]. Uncovering gender differences in the expression of a particular brain may help us understand gender differences in mental disorders.

First, we identified the brain structure that shows the greatest difference between genders. Specifically, for a specific brain structure $i$, we calculated the Euclidean distance between the representations of the structure for male $w_{i1}$and female $w_{i2}$ with the interaction representation $W$. Then, we selected the brain structure showing the greatest distance. The frontal cortex (BA9) showed the greatest distance and was chosen for further investigation.

After choosing the frontal cortex, we computed its adjusted gene expression profiles for both males and females by multiplying the submatrix of $W$ corresponding to the frontal cortex by the gene representation $V$ only on selected metagenes. In determining metagenes, we considered the top 2 metagenes showing the greatest difference between genders using the submatrix corresponding to the frontal cortex. Then, we examined the up- (Figure 2C) and down-regulated (Figure 2D) BPs enriched by the difference in the adjusted gene expression of the frontal cortex between genders.

Generally, the main theme of up- and down-regulated BPs (Figures 2C and 2D) for gender differences in expression of the frontal cortex are similar to those in Figures IIA and IIB. Further results regarding gender differences in BPs in BA9 can be found in the two figures. Note that the BA9 area is only one part of the frontal cortex, so the results may be biased and incomplete in revealing the whole picture of the frontal cortex.

To summarize, INSIDER demonstrates the following advantages over existing methods in analyzing the extracted GTEx data. It reduces dimension with respect to gender and brain regions. This task is inapplicable for GLM-based methods for DGE analysis. The downstream analyses with the outputs from INSIDER are also not unapplicable for conventional PCA- and NMF-based methods and tensor decomposition for the reasons discussed in the manuscript.

Figure 3 Supplement figures for analysis of GTEx


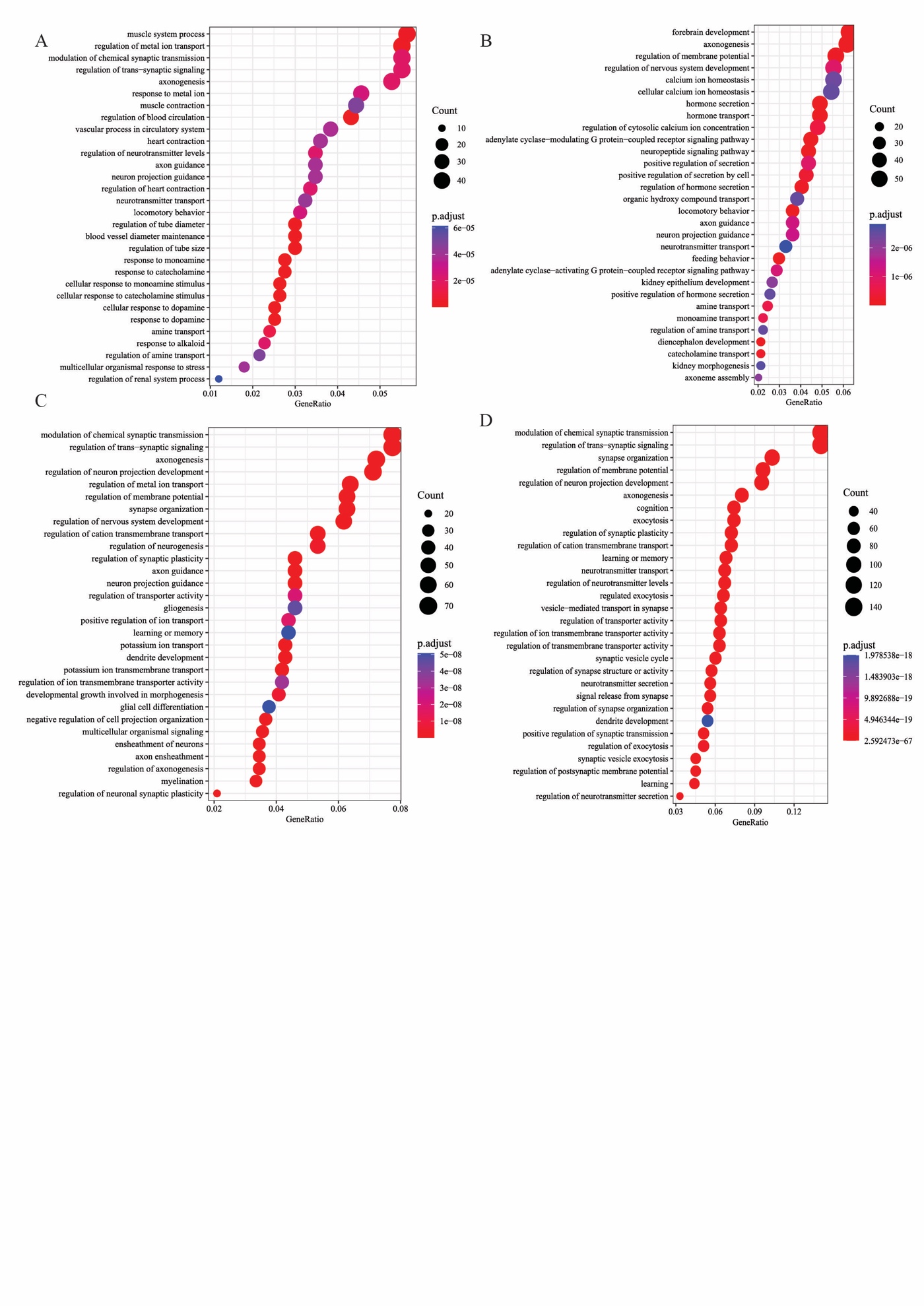


1. Figures A, B, C, and D show the top 30 up-regulated BPs enriched by the 3rd, 4th, 6th, and 12th metagenes, respectively.
2. **RCD algorithm for elastic net with screening rules**

| **Algorithm 1** RCD algorithm for elastic net with screening rules | | |
| --- | --- | --- |
| **Procedure** RCD$(U, Z, \lambda, \alpha, \beta, tol)$ | $\beta$ is the warm start, $tol$ is set to $1\times{10}^{-5}$ | |
| $r\leftarrow Z-U\beta$ | $r$ is the residual | |
| Find the set of indexes $s$ with Eq. 4 | Indexes in $s$ will be ignored in optimization | |
| $\beta\left[ s \right] \leftarrow0, \beta_{pre}\leftarrow0$, $l\leftarrow seq(0, length\left( \beta\right))$. |  |  |
| **While** $\left\vert\beta_{pre}-\beta\right\vert_{1}>tol$ **do** | end while if $\left\vert\beta_{pre}-\beta\right\vert_{1}\leq tol$ | |
| $\beta_{pre}\leftarrow\beta, l\leftarrow shuffle\left( l \right)$ |  |  |
| **foreach** $j$ in $l$ **do** |  |  |
| If $j$ in $s$; ***continue*** | | switch to the next iteration |
| Calculate $\beta_{k}^{new}$ with the below equation Eq. S1 | |  |
| $r\leftarrow r-\left( \beta_{j}^{new}-\beta_{j} \right)\times u_{j}$ | $u_{j}$ is the $j$-th column of $U$ | |
| $\beta_{j}\leftarrow\beta_{j}^{new}$ |  |  |
| **end for** |  |  |
| **End while** |  |  |
| **return** $\beta$ |  | the solution to the problem |
| **end procedure** |  |  |

### Derivation for random coordinate descent for elastic net problems

We define the objective function for the elastic net problem as

| $\mathcal{L}\left( \lambda,\beta\right)=\frac{1}{2}\left( Y-X\beta\right)^{T}\left( Y-X\beta\right)+\frac{1}{2}\left( 1-\alpha\right)\lambda\left\Vert\beta\right\Vert_{2}^{2}+\alpha{\lambda\left\vert\beta\right\vert}_{1}.$ |
| --- |

Here $Y$ is the outcome, $X$ is a $n\times p$ feature matrix, $\beta$ is the parameter vector, and $\alpha$ and $\lambda$ are regularization parameters. Here we derive the update for a single parameter $\beta_{j}$ for demonstration. Denote $\beta^{k}$ the parameter $\beta$ at the $k$-th iteration. We rewrite the above equation regarding $\beta_{j}$ for $k+1$ iteration as

$$\begin{matrix} \mathcal{L}\left( \lambda,\beta_{j} \right)= & \frac{1}{2}[\left( Y-X_{-j}\beta_{-j}^{k}-X_{j}\beta_{j} \right)^{T}\left( Y-X_{-j}\beta_{-j}^{k}-X_{j}\beta_{j} \right)]+ \\ & \lambda\left( \frac{1}{2}\left( 1-\alpha\right)\left( \left\| \beta_{-j}^{k} \right\|_{2}^{2}+\beta_{j}^{2} \right)+\alpha\left( \left| \beta_{-j}^{k} \right|_{1}+\left| \beta_{j} \right|_{1} \right) \right), \end{matrix}$$

where $X_{-j}$ is $X$ without the $j$-th column, and $\beta_{-j}^{k}$ is the $\beta^{k}$ with the $j$-th element removed and is known at the $k+1$ iteration when updating $\beta_{j}$. Taking the derivative of the above equation regarding $\beta_{j}$ and setting it to zero, we have

$\frac{\partial\mathcal{L}\left( \lambda,\beta_{j} \right)}{\partial\beta_{j}}=-X_{j}^{T}\left( Y-X\beta^{k} \right)-X_{j}^{T}X_{j}\beta_{j}^{k}+X_{j}^{T}X_{j}\beta_{j}+\lambda\left[ \left( 1-\alpha\right)\beta_{j}+\alpha\mathrm{sign}\left( \beta_{j} \right) \right]=0.$

The update for $\beta_{j}$ at $k+1$ iteration is as follows:

|  | $\beta_{j}=\frac{\mathrm{sign}\left( \mathbf{S}_{j} \right)\left( \left\vert\mathbf{S}_{j} \right\vert-\lambda\alpha\right)^{+}}{X_{j}^{T}X_{j}+\lambda\left( 1-\alpha\right)},$ | Eq. S1 |
| --- | --- | --- |

where $\mathbf{S}_{j}=X_{j}^{T}\left( Y-X\beta^{k} \right)+X_{j}^{T}X_{j}\beta_{j}^{k}$.

References

[1] J. Lonsdale *et al*, "The genotype-tissue expression (GTEx) project," *Nat. Genet.,* vol. 45, *(6),* pp. 580-585, 2013.

[2] G. Williams *et al*, "The hypothalamus and the control of energy homeostasis: different circuits, different purposes," *Physiol. Behav.,* vol. 74, *(4-5),* pp. 683-701, 2001.

[3] N. R. Eaton *et al*, "An invariant dimensional liability model of gender differences in mental disorder prevalence: evidence from a national sample." *J. Abnorm. Psychol.,* vol. 121, *(1),* pp. 282, 2012.
