# Supplementary figures and images for: INSIDER: Interpretable sparse matrix decomposition for RNA expression data analysis
Source: PLoS Genet. 2024 Mar 14;20(3):e1011189. doi: 10.1371/journal.pgen.1011189 (PMC10965063; doi:10.1371/journal.pgen.1011189)

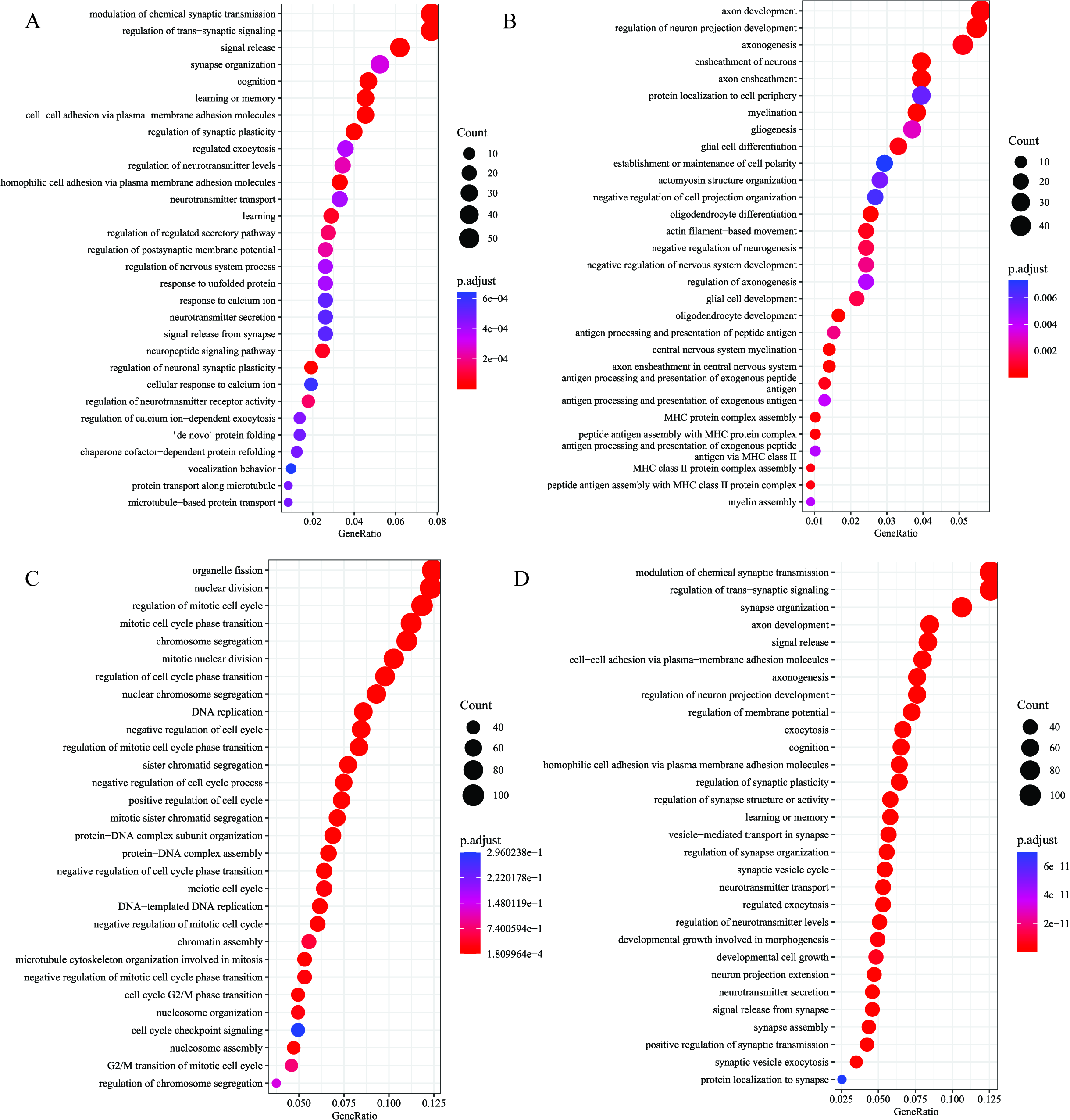

Supplement: S1 Fig — The top 30 up-regulated BPs (S1A Fig) enriched by the 2nd metagene and down-regulated BPs (S1D Fig) by the 17th metagene are related to learning, memory, cognition, and synaptic functions and neurotransmitter activities. The top 30 down-regulated BPs (S2B Fig) enriched by the 2nd metagene involve axon development, axonogenesis, myelination, and gliogenesis. The top 30 up-regulated BPs (S1C Fig) by the 17th metagene cover the BPs related to cell development and differentiation. (TIF) [file pgen.1011189.s002.tif]

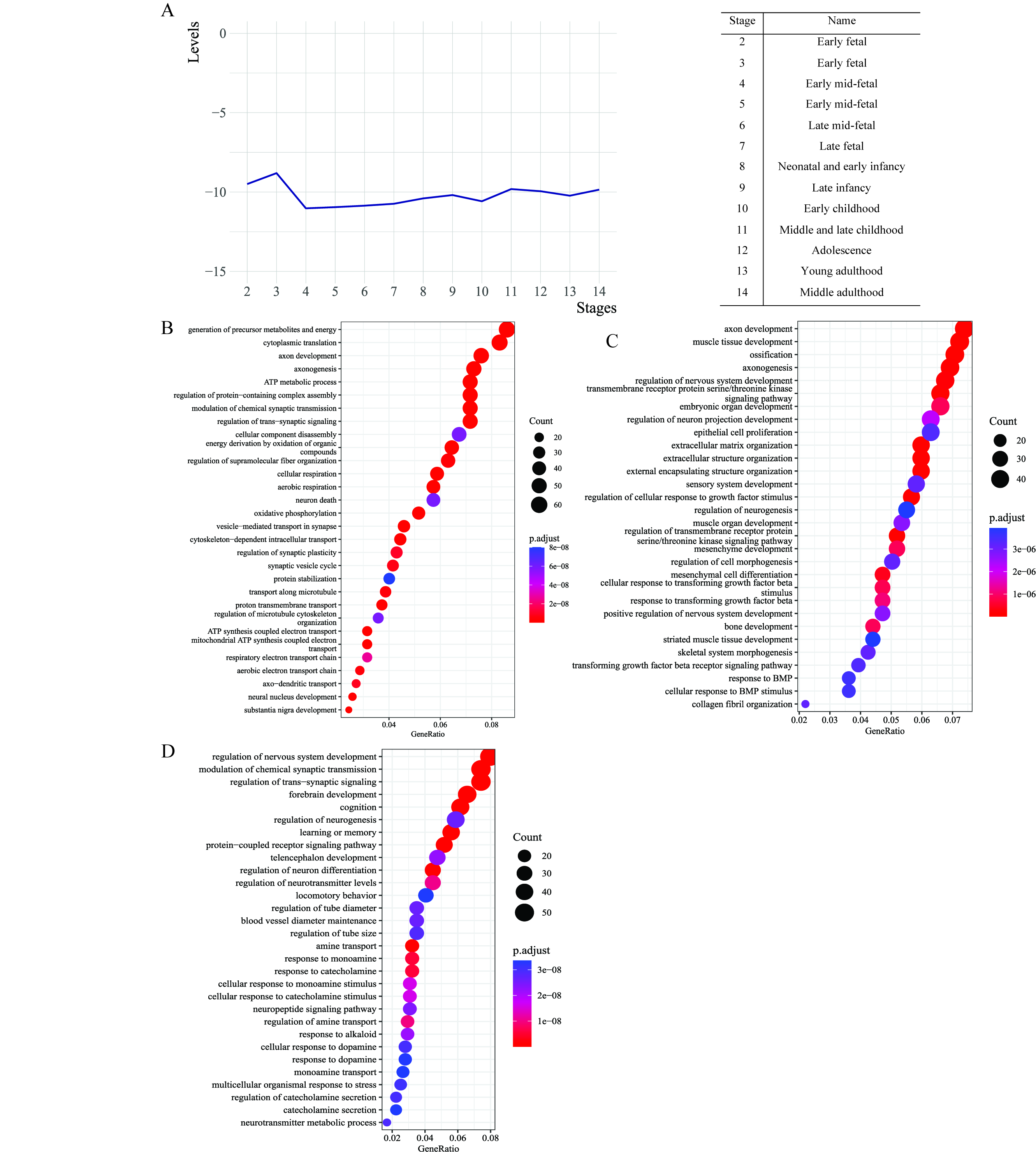

Supplement: S2 Fig — S2A Fig shows that the trajectory of the least variable metagene is negative and basically flat across human brain development. The top 30 down-regulated BPs (S2B Fig) encoded by the metagene involve cell and ATP metabolism and cell communication. Moreover, the top 30 up-regulated BPs (S2C Fig) enriched by the 5th metagene involve ossification and development of bone, muscle, and sensory systems, and the top 30 up-regulated BPs (S2D Fig) by the 1st metagene cover learning or memory, cognition, and hormone (e.g., dopamine, monoamine, and catecholamine) secretion and transport. (TIF) [file pgen.1011189.s003.tif]

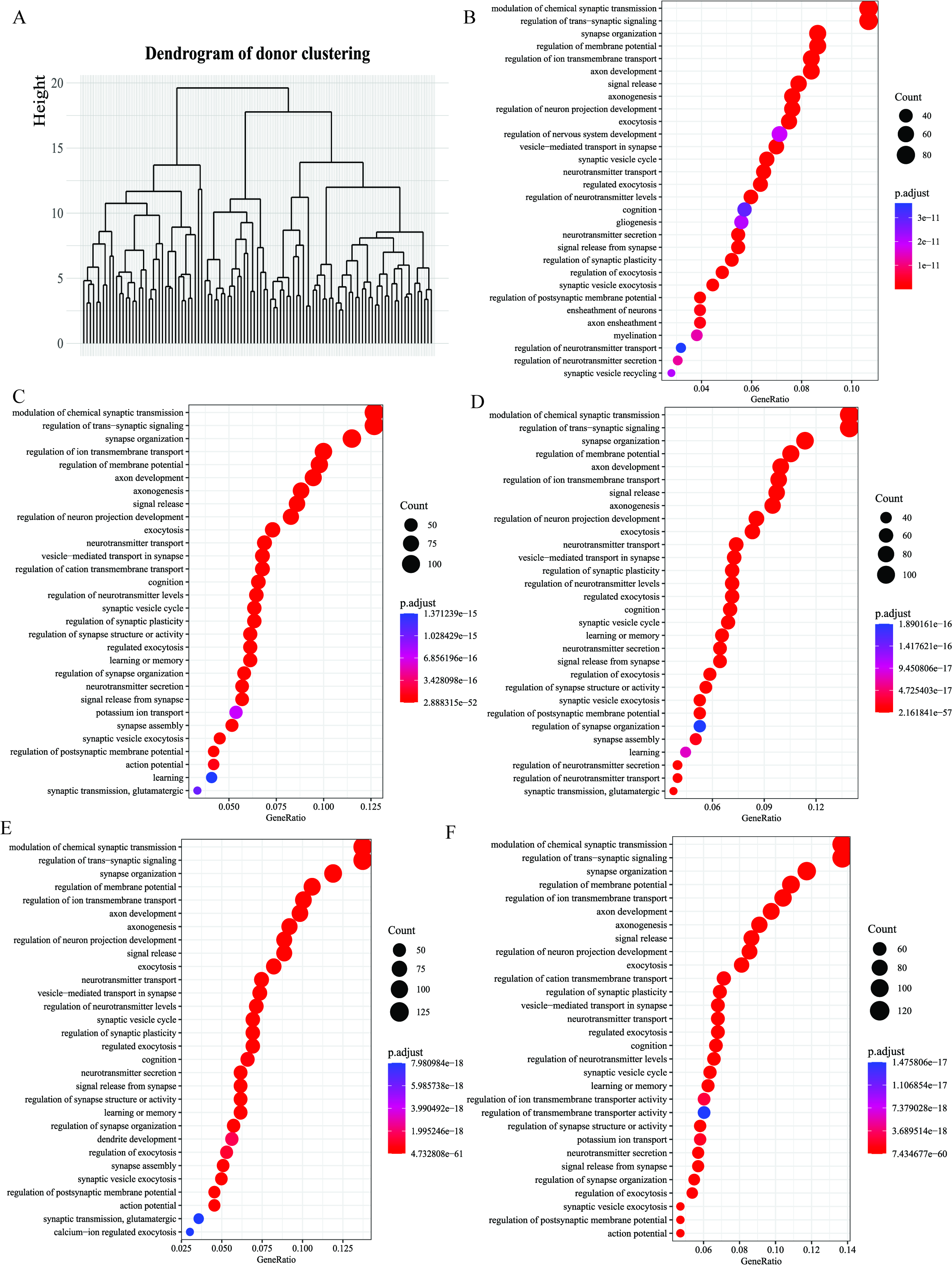

Supplement: S3 Fig — S3A Fig shows the dendrogram of hierarchical clustering on donor representation with the selected metagenes. We selected 3 subgroups for further analysis. The top 30 down-regulated BPs (S3B Fig) enriched by the 13th metagene involve cognitive functions, and the top 30 up-regulated BPs (S3C Fig) enriched by the 17th cover learning, memory, cognition, and synaptic functions. S3D Fig shows the top 30 down-regulated BPs enriched by the difference in gene expression of right HPC between dementia and control. Overall, the p-values for most down-regulated BPs in S3D Fig are significant at 2e-57, which are greater than those for the left HPC shown in Fig 3C from the manuscript (P at 1e-66). Moreover, the gene ratio in S3D Fig is also smaller than in Fig 3C. This leads to the finding that the left and right HPC may be affected differently by dementia. S3E and S4F Figs show the top 30 down-regulated BPs enriched by the difference in expression profiles for WM of the left and right forebrain, respectively. The BPs in S3E Fig are slightly more statistically significant than those in S3F Fig and the BPs in the two figures are basically the same. (TIF) [file pgen.1011189.s004.tif]

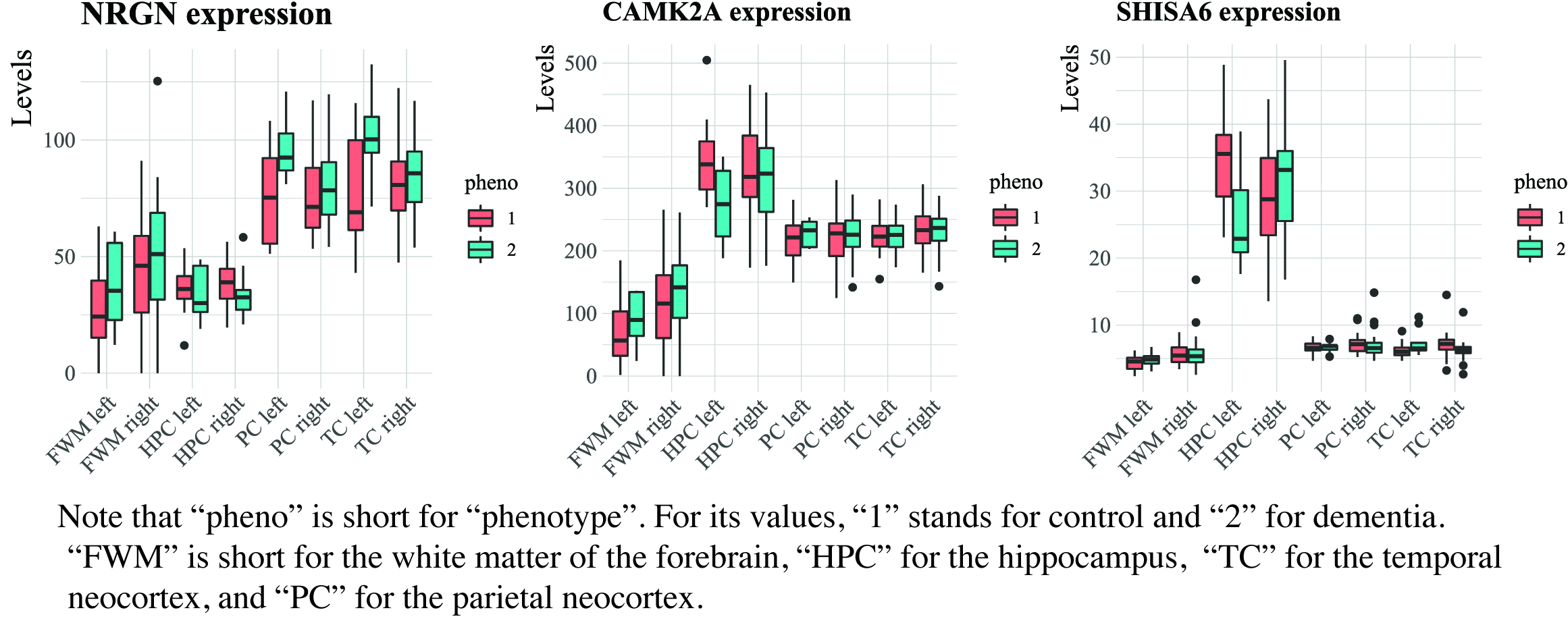

Supplement: S4 Fig — The figure compares the expression levels of three selected genes (NRGN, CAMK2A, and SHISA6) between dementia and control across brain regions, suggesting that dementia has potentially heterogeneous effects on the left and right HPC at gene levels. (TIF) [file pgen.1011189.s005.tif]
