# Supplementary material for: INSIDER: Interpretable sparse matrix decomposition for RNA expression data analysis
Source: PLoS Genet. 2024 Mar 14;20(3):e1011189. doi: 10.1371/journal.pgen.1011189 (PMC10965063; doi:10.1371/journal.pgen.1011189)
Supplement: Attachment — Submitted filename: Insider_response_letter_v4.docx [file pgen.1011189.s009.docx]

Reviewer #1:

This article proposed a dimension reduction approach for RNA expression data to decompose variation from different biological variables, which overcomes some limitations of the existing methods and was shown to be useful in the real data analysis. My comments are as follows.

1. It is still not very clear to me how to interpret the metagenes together with the covariates, especially the matrix V. Because it seems that V has nothing to do with the covariates, but the metagenes from INSIDER would be different under the models with different covariates. Also, what is the difference in the interpretation of the metagenes obtained from INSIDER compared to those obtained from PCA or factor analysis?

We thank the reviewer for the insightful comment. In INSIDER, the variation of $Z^{N\times M}$ is decomposed into $(X_{D}D+X_{P}P+X_{T}T+X_{W}W)V$, where $X_{D}^{N\times N1},X_{P}^{N\times N_{2}},X_{T}^{N\times N_{3}},X_{W}^{N\times(N_{2}*N_{3})}$ are the indicator matrices for donors, phenotypes, tissues, and phenotype by tissue interactions, $D^{N_{1}\times K}$, $P^{N_{2}\times K}$, $T^{N_{3}\times K}$, $W^{{(N}_{2}*N_{3})\times K}$ are the matrices for the effects of donors, phenotypes, tissues, and phenotype by tissue interactions on $K$ latent metagenes, and $V^{K\times M}$ represents represents the effects of the $K$ metagenes on the expression of $M$ individual genes. This decomposition assumes that the covariates represented by $X_{D}$, $X_{P}$, $X_{T}$, and $X_{W}$ affect activities of transcription factor (TFs) or other biological processes/signals, represented by metagenes, which in turn affect the expression of groups of genes. Crucially, our decomposition assumed that the covariates are correlated with gene expression levels only through their effects on the latent metagenes.

Regarding the difference in the interpretation of INSIDER and PCA or factor analysis, we would like to use factor analysis for illustration. Usually, factor analysis is formulated as $Z^{N\times P}=F^{N\times K}L^{K\times P}+\Theta^{N\times P}$ with the restrictions that the columns of $F$ be uncorrelated and of unit variance and that the columns of the error matrix $\Theta$ be independent. Here each row of $Z$ denotes an observation with $P$ variables, $L^{K\times P}$ is the loading matrix, and $F^{N\times K}$ is the factor matrix.

Regarding the difference in the interpretation of INSIDER and PCA or factor analysis (FA), we note that both PCA and FA can be formulated as a decomposition of the data matrix. FA can be formulated as $Z^{N\times P}=F^{N\times K}L^{K\times P}+\Theta^{N\times P}$ with the restrictions that the columns of $F$ be uncorrelated and of unit variance and that the columns of the error matrix $\Theta$ be independent. Here each row of $Z$ denotes an observation with $P$ variables, $L^{K\times P}$ is the loading matrix, and $F^{N\times K}$ is the factor matrix. Each factor (column of $F$) contributes to two or more variables according to the corresponding row of loading matrix $L^{K\times P}$, and induces covariances among these variables. Here INSIDER can be considered a generalization/extension of factor analysis, where the factors are modelled as being under the influence of some covariates. In INSIDER, we introduce a model of $F$ in terms of covariates $X$ by $F=XB$, where $X$ is an indicator matrix of measured covariates and $B$ is a matrix of coefficients. The covariates are classified into donor ($X_{D}$), phenotype ($X_{P}$), tissue ($X_{T}$), and phenotype-by-tissue interactions ($X_{W}$). However, these indicator matrices can be joined into a single matrix $X=(X_{D}, X_{P}, X_{T}, X_{W})$, if the coefficient matrices are also joined together into a single matrix $B={(D^{⊺}, P^{⊺}, T^{⊺}, W^{⊺})}^{⊺}$. Thus, this gives the overall INSIDER model by $Z=XBV+\Theta$. Therefore, in INSIDER, a metagene is analogous to a factor, while the matrix *V* is analogous to the loading matrix.

Different from factor analysis, INSIDER directly associates the covariates ($X_{D}$, $X_{P}$, $X_{T}$, $X_{W}$) with the metagenes through the matrices $D,P,T,W$, which provides insights on how covariates affect the biological pathways. Let us use the brain region representation $T^{N_{3}\times K}$ as an example for illustration. For example, compared with other brain regions, the striatum has a highly positive value in the first column of $T$ (the expression levels of the 1st metagenes across different brain regions), then enrichment analysis with the first row of $V$ (the 1st metagene) can help to characterize the function of the brain region. However, this analysis is inapplicable to factor analysis because the estimation of the factors is not guided by the covariates and thus it cannot directly associate the factors with the covariates. In addition, INSIDER enables computing adjusted expression profiles for covariates to uncover the effect of covariates on gene expression. For example, in our second application, the biological mechanism of dementia is uncovered by examining down-regulated BPs enriched by the difference in adjusted gene expression between dementia and control. This is also inapplicable to factor analysis.

We have added some contents at the Discussion section to discuss the difference in interpretation between INSIDER and PCA/factor analysis.

2. The article mentioned that “INSIDER decomposes variation from different biological variables”, but only the discrete covariates have been discussed. Is INSIDER also able to handle the continuous covariates?

We thank the reviewer for the comment. INSIDER can handle the continuous covariates in the following ways.

Firstly, we can discretize the continuous covariate into several categories. The strategy for the discretization depends on the research interest and study aim. After the discretization, we may apply INSIDER to obtain latent representations for the covariate categories. Here we use the BrainSpan data as an example. The study [1] defines development stages by discretizing the age distribution of donors into several categories, and INSIDER can compute latent representation for each category of development stage.

Secondly, INSIDER also can be directly extended to incorporate continuous covariates as follows. For illustration, let denote $q$ is a vector of length $K$ for latent representation for a continuous covariate, and $x_{q}^{N\times1}$ is the variable across $N$ observations.

$${\mathcal{L(}D, q,T,W,V)}=\frac{1}{2}\left\| Z-\left( X_{D}D+x_{q}q^{⊺}+X_{T}T+X_{W}W \right)V \right\|_{F}^{2}+$$

$\frac{1}{2}\lambda\left[ \left\| D \right\|_{F}^{2}+\left\| q \right\|_{2}^{2}+\left\| T \right\|_{F}^{2}{+\left\| W \right\|}_{F}^{2} \right]+$

$\lambda\left[ \frac{1}{2}\left( 1-\alpha\right)\left\| V \right\|_{F}^{2}+\alpha\left| V \right|_{1} \right]$.

In the equation, the meaning of other notations is the same as in the manuscript. With other parameters fixed, the objective function with respect to $q$ can be simplified as follows:

$$\mathcal{L}\left( q \right)=\mathrm{argmin}_{q} \frac{1}{2}\mathrm{Tr}\left[ \left( \tilde{Z}-x_{q}q^{⊺}V \right)^{⊺}\left( \tilde{Z}-x_{q}q^{⊺}V \right) \right]+\frac{1}{2}\lambda\left\| q \right\|_{2}^{2},$$

where $\tilde{Z}=Z-\left( X_{D}D+X_{T}T+X_{W}W \right)V$. By minimizing the objective with respect to $q$, the update for $q$ can be obtained by

$q=\left( x_{q}^{⊺}x_{q}VV^{⊺}+\lambda\mathbb{I}_{k} \right)^{-1}V\tilde{Z}^{⊺}x_{q}$.

To further improve the generality of INSIDER, we have extended it by incorporating the above content to the subsection “Optimize the objective function” and updating our software accordingly.

3. In the real data analysis of BrainSpan data, development stages and brain regions are used as covariates. However, the development stages are in chronological order. Does INSIDER handle the ordinal variables and the unordered categorical variables in the same manner?

We thank the reviewer for the insightful comment. In the analysis of BrainSpan data, the variation of developmental stages is modeled by $X_{D}^{N\times N_{1}}D^{N_{1}\times K}V^{K\times M}$, where $X_{D}^{N\times N_{1}}$ represents the covariates of developmental stages, which is the dummy indicator matrix for developmental stages, $D^{N_{1}\times K}$ is the expression levels of $K$ metagenes across $N_{1}$ developmental stages, and $V$ contains the information for metagenes. Here we consider development stages as unordered categorical variables for the following reasons.

On the one hand, human brain development is nonlinear, so some metagenes may only be highly active in certain developmental stages and then are leveled down or turned off. For example, the study [2] systematically discussed the development of different brain structures across developmental stages, suggesting that the development of brain structures is specific to certain development stages. It is more reasonable to model development stages as unordered categorical variables.

On the other hand, we carried out simulation studies to show INSIDER can recover the monotonic trend for ordinal variables in the revision. In our simulation setup, we consider a gene expression matrix $Z$ (250×200), and variation in the matrix comes from two sets of covariates ($X_{E},X_{F}$), which are dummy matrices for two categorical variables, their interaction, and random noise. Their relationship is described by $Z=T+\Theta$, where $T=\left( X_{E}E+X_{F}F+X_{R}R \right)V$. The rank of latent space is set to 5, $E$ and $F$ are matrices of dimensions 50×5 and 5×5, respectively, and the dimension of $R$ for the interaction between the two sets of covariates is 250×5. $V$ is a 5×250 matrix for gene latent representations. Here $X_{F}$ contains an ordinal variable with 5 levels, and the $i$-th row of $F^{5\times5}$ is generated from $N\left( i, 0.25 \right),\forall i=1,\ldots,5$, so $F$ is monotonic increasing across the 5 levels. All other matrices were generated from the standard normal distribution. The design matrices for $\{E, F,R\}$ are $X_{E}^{250\times50}$, $X_{F}^{250\times5}$, and $X_{R}^{250\times250}$, respectively. The noise matrix $\Theta$ (250×200) was generated from a normal distribution $N(0,\delta)$, and $\delta$ is the variance of the distribution. We considered $\delta$ equal to 0.1, 0.2, 0.3, 0.4, and 0.5 in our simulations. The result of the above simulation is presented in the table below. From the table, INSIDER well recovered the monotonic trend of the ordinal variable $F$.

| Noise level | Ordinal variable $F$ | Level 1 | Level 2 | Level 3 | Level 4 | Level 5 |
| --- | --- | --- | --- | --- | --- | --- |
| 0 | True mean of row of $F$ | 0.9541 | 1.9988 | 3.0409 | 3.9389 | 5.0232 |
| 0.1 | Row mean of estimated $\hat{F}$ by INSIDER | 0.7350 | 1.7280 | 2.9561 | 3.7810 | 4.9520 |
| 0.2 |  | 0.7637 | 1.8268 | 3.0020 | 3.8456 | 4.9192 |
| 0.3 |  | 0.7567 | 1.8618 | 2.9883 | 3.8964 | 4.9510 |
| 0.4 |  | 0.7653 | 1.8421 | 3.0437 | 3.8846 | 4.9799 |
| 0.5 |  | 0.7538 | 1.8587 | 3.0131 | 3.9384 | 5.0150 |

Table 1. The true and estimated mean for the 5 levels of the ordinal variable $F$ over the 5 latent variables

In summary, it is a feasible solution for INSIDER to consider ordinal variables as unordered categorical variables. We also would like to thank the reviewer for pointing out a good future direction for INSIDER to consider ordinal variables explicitly.

We added some content in the 3rd paragraph of the section “Analysis of the BrainSpan data” to justify our practice and in the Discussion section to discuss this issue.

4. In the section “Metagenes reveal development trajectories”, it said “The expression level of the most variable metagene is high before late fetal stages and decreases to a low level after early childhood”. I wonder how the expression level of the metagene is calculated. Is there an explicit formula of the metagene? Could the author give more details?

We thank the reviewer for the comment. In the real data application of BrainSpan data, the variation of developmental stage is modeled by $X_{D}^{N\times N_{1}}D^{N_{1}\times K}V^{K\times M}$. Here $X_{D}$ is the covariate for developmental stages. Rows of $V$ are metagenes, capturing genes that that co-express or work together to induce biological functions. $D^{N_{1}\times K}$ are the levels of expression of $K$ metagenes across $N_{1}$ developmental stages. In practice, we compute the variance of the expression levels of each metagene across $N_{1}$ developmental stages (the variances are computed with the columns in $D$) and select the metagene with the greatest variance, which is the most variable metagene. For illustration, suppose the most variable metagene is the 2nd metagene, and $d_{2}$ denotes the second column of $D$. The expression levels of the 2nd metagene across development stages correspond to the values of $d_{2}$. Here we investigate changes in the values of $d_{2}$ for different developmental stages. In practice, the values of $d_{2}$ for the stages before late fetal are high, and the values of $d_{2}$ decrease to a low level after early childhood. This leads to the statement mentioned in the question.

We also added more details in the first paragraph of the subsection “Metagenes reveal development trajectories” to improve the clarity.

5. In the enrichment analyses for a metagene, say the 2nd metagene, it is not clear to me how to conduct the analyses because the metagene is a latent variable. It would be beneficial if more details could be provided.

We thank the reviewer for the comment. Specifically, for a given metagene $V_{i}$, the $i$-th row of $V$, we examine the biological processes (BPs) enriched by genes with large effects, that is, the genes with large absolute values for $V_{i}$, since these genes encode primary biological functions of the metagene. Therefore, enrichment analyses were conducted on the set of genes in the upper or lower 2.5% quantile of $V_{i}$ to explore up- or down-regulated BPs encoded by the metagene, respectively. In the implementation, the function *enrichGO* from the R package *clusterProfiler* (v4.4.4) was utilized, with the “*ont*” parameter set to "BP".

We have revised the second paragraph of the subsection “Metagenes reveal development trajectories” in our manuscript to provide more details on the enrichment analysis.

Reviewer #2:

Dimension reduction is critically important in data exploration analysis. Particularly it provides the stepstone to understand the biological signals in high dimensional RNA-seq data. In this paper, the authors propose INSIDER, a general and flexible statistical framework based on matrix factorization. INSIDER decomposes variation from different biological variables and their interactions into a shared low-rank latent space. Particularly, it introduces the elastic net penalty to induce sparsity while considering the grouping effects of genes. It can achieve dimension reduction of high-dimensional data (of >=3 dimensions), as opposed to conventional methods (e.g., PCA/NMF) which generally only handle 2D data (e.g., sample ´ expression). Besides, it enables computing 'adjusted' expression profiles for specific biological variables while controlling variation from other variables.

Overall, the idea is novel and to develop new method to explain the data. better. INSIDER is a general and flexible framework. It can capture interactions between variables or covariate and encourages sparsity in latent representations by imposing the elastic net penalty. The advantages have been highlighted. The paper is well written and the motivation is explained quite clear.

My minor concern is as follows,

1. To use INSIDER well, the users have to know the major variation sources. For example, it’s quite typical for expression data with donors, phenotypes, tissues information. But sometimes those information is not available. How should the method be used?

We thank the reviewer for the insightful comment. It helps us improve the generality of INSIDER further. As presented in the manuscript, INSIDER aims for dimension reduction with respect to known covariates. In our work, we used donors, phenotypes, and tissue as examples for illustration. However, when donor, phenotype, and tissue information are unavailable, our method can be extended to model variation from unknown sources by introducing $U$. Thus, in this situation the objective function for INSIDER is as follows:

$${\mathcal{L(}D, P,T,W,U,V)}=\frac{1}{2}\left\| Z-\left( X_{D}D+X_{P}P+X_{T}T+X_{W}W+X_{U}U \right)V \right\|_{F}^{2}+$$

$\frac{1}{2}\lambda\left[ \left\| D \right\|_{F}^{2}+\left\| P \right\|_{F}^{2}+\left\| T \right\|_{F}^{2}{+\left\| W \right\|}_{F}^{2}+\left\| U \right\|_{F}^{2} \right]+$

$\lambda\left[ \frac{1}{2}\left( 1-\alpha\right)\left\| V \right\|_{F}^{2}+\alpha\left| V \right|_{1} \right]$

Here $X_{U}$ is an identity matrix, which can be ignored and is added in consideration of consistency in notations, and $U^{N\times K}$ captures variation from unknown sources. The meanings of other notations are the same as in the manuscript. This above equation is equivalent to introducing one variable with each observation belonging to a unique category. Regarding optimizing the objective, the same strategy is utilized.

We updated the sections “Overview of INSIDER” and “Methods” to accommodate the extension of INSIDER. In the manuscript, we mentioned that INSIDER is flexible and that the covariates in INSIDER can be adjusted according to the research design and study aim. In the case that sources of variations are not available, INSIDER can be adjusted by ignoring the unavailable covariates and include $U$ to capture variation from unknown sources. We have also updated the code and documentation of our software in GitHub accordingly to accommodate this situation in our implementation.

2. The authors added the phenotype by tissue interactions as an example. To make the method more general, it’s better to having one version without interaction term. It’s also good to demonstrate how the interaction term improves the results.

We thank the reviewer for the helpful suggestion to generalize INSIDER further. To improve the flexibility and generality of INSIDER, we updated its software to allow freely include or exclude covariates and/or interaction terms in data analysis with INSIDER. In the manuscript, we also emphasize that covariates and interactions in INSIDER can be included or excluded according to the research design and study aim. We observed the benefits of incorporating phenotype by tissue interactions from the following two aspects:

1) This practice improves the interpretability of INSIDER. First, incorporating the interactions enables us to reveal the heterogeneous effects of one variable (e.g., dementia) on another variable (brain structures). Here we illustrate with the application to the ageing, dementia, and TBI data as an example. In the application, we considered dementia by brain structure interaction to model the variation of dementia on different brain structures. Then, further downstream analysis with enrichment analysis shows that the left hippocampus is more vulnerable to dementia than the right hippocampus, which is consistent with previous studies. Details are presented in the manuscript. In short, the interaction term of INSIDER can help us understand the interactive effect between covariates in gene expression. This is ignored by conventional methods, such as PCA, NMF, factor analysis, and their variants.

2) To further verify whether the performance of INSIDER is significantly improved by considering interaction, we conducted the following statistical test. Generally, performance on held-out data is considered a golden standard to evaluate model performance. Thus, we examine whether incorporating interaction terms improves the performance of INSIDER on held-out data with root-mean-square error (RMSE). First, we randomly drew 10% of the elements without replacement from the data matrix as testset using a fixed random seed. Second, we followed the same procedure in the manuscript to select hyperparameters for INSIDER with and without the interaction term. Then, we ran INSIDER with selected hyperparameters 50 times for 30 iterations with different parameter initializations and reported their RMSE on the held-out data. Finally, we examined whether the RMSE of INSIDER with interaction is statistically significantly lower than that of INSIDER without interaction with a one-sided t-test. The *p*-value (*p*-value < 2.2e-16) from the test suggests that incorporating interaction improves the performance of INSIDER. The mean with standard error for INSIDER with and without interaction are 0.2297+0.0001 and 0.2302+0.0001, respectively. From our understanding, the relatively small improvement in RMSE is due to the fact that only a small proportion of genes play a role in the interaction between dementia and brain structures.

We added a new subsection “Incorporating the interaction improves INSIDER” in the manuscript to demonstrate how the interaction term improves the results.

3. To highlight the high-dimensional data (of >=3 dimensions), it’s better to have more examples to show high-dimensional data is common and quite general case in practice.

We thank the reviewer for the valuable suggestion. To show the wide applicability of INSIDER, we have added another example with GTEx data. In the application, we explored the gender difference in gene expression across different brain structures. The results of the analysis are provided below and are added to the manuscript. Meanwhile, we also highlight the availability of high-dimensional data sources with the same examples in the Introduction of the manuscript.

## Analysis of gender differences in gene expression of human brain with GTEx

In this application, we seek to explore gender differences in gene expression across 13 different brain regions from the GTEx data [3]. Details of data processing are shown in the Supplementary Content. The dimension of our processed data is 988×49,999 for samples from 318 different donors. For each brain region, it has 38 RNA-seq samples from either men or women. We modeled three covariates: brain region, gender, and gene. The number of ranks $K$ of latent space selected by hyperparameter tuning is 12.

### Metagenes reveal gender differences in gene expression of human brain

To reveal gender differences in gene expression of human brain, we calculated the adjusted gene expression profiles for genders by multiplying gender representation with gene representation and then examined up- (Figure IIA) and down-regulated (Figure IIB) BPs enriched by the difference in gene expression between the male and the female.

On the one hand, expression levels of genes enriched for synapse activities and neurotransmitters are higher in men than in women across the brain regions included (Figure IIA). On the other hand, women have higher expression levels of genes contributing to immune responses, such as (defense) response to viruses and bacterium and humoral immune response, than men (Figure IIB).

### Metagenes characterize functions of brain regions

Previously, we revealed gender differences in gene expression of human brain with metagenes. Additionally, metagenes also enable us to uncover the functions of brain regions included. Intuitively, if the loading of a specific metagene for one brain region is significantly greater than that of other brain regions, then the metagene can be utilized to characterize the brain region. Following the intuition, we highlighted several metagenes (Table S3 in the Supplementary Tables) to demonstrate how to achieve this goal in practice.

Figure II Downstream analyses with results from the application to the GTEx data

1. Figures A and B show the top 30 up- and down-regulated BPs enriched by the difference in gene expression between genders, respectively.
2. Figures C and D display the top 30 up- and down-regulated BPs enriched for the difference in gene expression of the frontal cortex (BA9) between genders, respectively.

First, we found that the loading of the 3^rd^ metagene is highly positive and much greater in the three subcortical nuclei (putamen, nucleus accumbens, and caudate) than in other brain regions (Table S3). The top 30 BPs enriched by up-regulated genes of the metagene mainly fall into the following aspects: muscle contraction and system process, blood circulation and circulatory system, response to monoamine, catecholamine, and dopamine, synaptic transmission and signaling, and neurotransmitter transport (Figure IIIA), highlighting their roles in human motor and emotions.

Meanwhile, we also examined the top 30 up-regulated BPs enriched by the 4^th^ (Figure IIIB) and 6^th^ (Figure IIIC) metagenes since the loadings of the 4^th^ metagene for the hypothalamus and 6^th^ metagene for HPC are highly positive and much greater than for other brain regions (Table S3). The up-regulated BPs enriched by the 4^th^ metagene primarily involve hormone secretion and transport, calcium ion homeostasis, and catecholamine and monoamine transport (Figure IIIB), which is consistent with the role of the hypothalamus in homeostasis [4]. Meanwhile, the up-regulated BPs enriched by the 6^th^ metagene involve learning and memory, synaptic transmission and signaling, and dendrite development (Figure IIIC), revealing the key functions of HPC.

Moreover, we also explored the top 30 up-regulated BPs enriched by the 12^th^ metagene, whose loading is highly negative in the spinal cord. The up-regulated BPs involve the following biological activities: learning, memory, cognition, synaptic transmission and signaling, and neurotransmitter transport and secretion (Figure IIID), implying a much lower involvement of the spinal cord in human cognitive function, compared with other brain regions.

Figure III Supplement figures for analysis of GTEx

1. Figures A, B, C, and D show the top 30 up-regulated BPs enriched by the 3rd, 4th, 6th, and 12th metagenes, respectively.

### Explore gender differences in the expression of specific brain regions

In the previous subsection, we revealed gender differences in gene expression of human brain across all brain regions included. Still, psychiatrists might be more interested in the gender difference in a specific brain region since genders show differences in mental disorder prevalence [5]. Uncovering gender differences in the expression of a particular brain may help us understand gender differences in mental disorders.

First, we identified the brain structure that shows the greatest difference between genders. Specifically, for a specific brain structure $i$, we calculated the Euclidean distance between the representations of the structure for male $w_{i1}$and female $w_{i2}$ with the interaction representation $W$. Then, we selected the brain structure showing the greatest distance. The frontal cortex (BA9) showed the greatest distance and was chosen for further investigation.

After choosing the frontal cortex, we computed its adjusted gene expression profiles for both males and females by multiplying the submatrix of $W$ corresponding to the frontal cortex by the gene representation $V$ only on selected metagenes. In determining metagenes, we considered the top 2 metagenes showing the greatest difference between genders using the submatrix corresponding to the frontal cortex. Then, we examined the up- (Figure IIC) and down-regulated (Figure IID) BPs enriched by the difference in the adjusted gene expression of the frontal cortex between genders.

Generally, the main theme of up- and down-regulated BPs (Figure IIC and IID) for gender differences in expression of the frontal cortex are similar to those in Figure IIA and IIB. More detailed gender differences in BPs in BA9 can be found in the two figures. Note that the BA9 area is only one part of the frontal cortex, so the results may be biased and incomplete in revealing the whole picture of gender differences in gene expression of the frontal cortex.

We have added the above content into the section S7 of our Supplementary Content.

4. The choice of K is a multi-scale problem. How should we choose it in practice.

We thank the reviewer for the comment. Here are the details for the choice of $K$. In practice, we first randomly draw 10% of elements without replacement from the data matrix as testset and then treat the kept data as trainset. Only the trainset is used to train INSIDER, and the testset is independent from model training and utilized for model selection. Specifically, we first train INSIDER on the trainset and then examine whether our approximations for the missing entries by the trained model can well approximate the observed values in the test set. This practice is also consistent with the idea of cross-validation, which performs model selection based on model performance on held-out data. In fact, it is also widely used in recommendation systems [6] and matrix completion [7].

To evaluate the performance of INSIDER with different choices of $K$, the root mean squared error (RMSE) on the testset is calculated, which measures the averaged squared difference between the approximations from INSIDER and the true values, so a lower RMSE on the testset corresponds to a better model. In our applications, $K$ was chosen from a sequence from 10 to 30 with step size 2. The range of $K$ is enough in general and can be broadened to consider more complex models. For each try of $K$ from the sequence, we run INSIDER for 30 iterations with hyperparameters $\lambda=0.1$ and $\alpha=0$ and report RMSE on the testset. Then, we select the rank $K$ corresponding to the lowest RMSE on the testset. For demonstration with the BrainSpan dataset, we applied the above procedure and showed the RMSE on the trainset and testset for the rank $K$ chosen from 3 to 50 with step size 2, presented in the figure below. The figure shows that the RMSE on the trainset decreases with increasing rank $K$, but the RMSE on the testset decreases at the beginning and increases after the complexity of INSIDER is higher than needed. In practice, we choose the rank $K$ corresponding to the lowest RMSE on the testset.

Figure 1. RMSE on the trainset and testset for different choices of $K$ in application to the BrainSpan data

Also, we examined the effect of different numbers of iterations (e.g., 30, 50) on model selection. We found that 30 iterations are sufficient to give consistent selection results, compared with more iterations (50 iterations). For demonstration, we show RMSE on the trainset and testset with 30 and 50 iterations for the rank $K$ chosen from 11 to 50 with step size 2, and the results are presented in Figure 2 and Table 1.

Figure 2. RMSE on the trainset and testset for different choices of $K$ after 30 and 50 iterations in application to the BrainSpan data

The above figure and table show that choosing $K$ with 30 and 50 iterations gives highly consistent results in terms of RMSE. Previous studies [8], [9] also used a small number of iterations (e.g., 20 iterations) to select the rank $K$ for methods based on matrix factorization. Note that our method for selecting rank $K$ is quite computationally efficient, since we set $\alpha=0$ when we select $K,$ and the update for all parameters can be computed in a closed form.

Table 1. RMSE on the trainset and testset for different choices of $K$ after 30 and 50 iterations in application to the BrainSpan data

| Rank *K* | Trainset  (30 iterations) | | Testset  (30 iterations) | Trainset  (50 iterations) | Testset  (50 iterations) |
| --- | --- | --- | --- | --- | --- |
| 3 | 0.40209 | 0.40450 | | 0.40209 | 0.40450 |
| 5 | 0.37800 | 0.38169 | | 0.37800 | 0.38169 |
| 7 | 0.36429 | 0.36994 | | 0.36429 | 0.36994 |
| 9 | 0.35457 | 0.36200 | | 0.35457 | 0.36200 |
| 11 | 0.34810 | 0.35686 | | 0.34810 | 0.35686 |
| 13 | 0.34450 | 0.35479 | | 0.34450 | 0.35479 |
| 15 | 0.34182 | 0.35330 | | 0.34182 | 0.35330 |
| 17 | 0.33992 | 0.35273 | | 0.33992 | 0.35273 |
| 19 | 0.33842 | 0.35261 | | 0.33838 | 0.35256 |
| 21 | 0.33713 | 0.35274 | | 0.33713 | 0.35274 |
| 23 | 0.33628 | 0.35320 | | 0.33628 | 0.35321 |
| 25 | 0.33571 | 0.35397 | | 0.33571 | 0.35397 |
| 27 | 0.33534 | 0.35498 | | 0.33534 | 0.35498 |
| 29 | 0.33503 | 0.35604 | | 0.33503 | 0.35609 |
| 31 | 0.33477 | 0.35723 | | 0.33477 | 0.35725 |
| 33 | 0.33454 | 0.35829 | | 0.33454 | 0.35830 |
| 35 | 0.33435 | 0.35968 | | 0.33434 | 0.35971 |
| 37 | 0.33417 | 0.36135 | | 0.33417 | 0.36159 |
| 39 | 0.33412 | 0.36155 | | 0.33412 | 0.36138 |
| 41 | 0.33412 | 0.36140 | | 0.33412 | 0.36128 |
| 43 | 0.33412 | 0.36149 | | 0.33412 | 0.36131 |
| 45 | 0.33412 | 0.36144 | | 0.33412 | 0.36130 |
| 47 | 0.33412 | 0.36134 | | 0.33412 | 0.36121 |
| 49 | 0.33412 | 0.36144 | | 0.33412 | 0.36127 |

We revised the corresponding content in the section “Initialization, hyperparameter tuning and the stopping criterion” in the manuscript to improve the clarity and justifiability of our method in selecting the rank $K$.

5. It’s better to provide software in GitHub

We thank the reviewer for the suggestion. We have released the software in GitHub and also provided the link for the software (https://github.com/kai0511/insider) in the abstract part of the manuscript.

From the editorial board:

Two reviewers and the editorial team have reviewed the manuscript. They showed overall enthusiasm for the proposed method as a general and principled approach to dimension reduction, while also offering several suggestions as follow. Please carefully review those questions and suggestions and respond to them accordingly.

1. Firstly, the authors claim that the INSIDER method can manage missing data. However, this aspect isn't explored in depth. The effect of significant and complex missing data remains uncertain. It would be beneficial to conduct further evaluations through simulation studies.

We thank the editor for the constructive comment. There are mainly two types of missing in RNA-Seq studies. The first type is missing samples. For example, the biosamples of some tissues from donors are not available, and this leads to missing samples in RNA-Seq data. The second type is missing entries. That is, expression levels of some genes for samples are missing. In practice, the proportion of missing entries is usually small in RNA-Seq data. Here we studied the two types of missing in RNA-Seq studies.

In our simulation study, we assume there are 50 donors, 8 tissues, and 1000 genes. The number of samples for our simulated data matrix is 400 (50×8). Moreover, we consider the interaction between phenotypes and tissues. In our simulation setup, we consider a gene expression matrix $Z$ (400×1000), and variation in the matrix comes from three biological variables ($D,P, T$), the interaction between $P$ and $T$, and random noise. Their relationship is described by $Z=R+\Theta$, where $R=\left( X_{D}D+X_{P}P+X_{T}T+X_{W}W \right)V$. The rank of latent space is set to 5, $D$, $P$, and $T$ are matrices of dimensions 50×5, 2×5, and 8×5, respectively, and the dimension of $W$ for the interaction between $P$ and $T$ is 16×5. $V$ is a 5×1000 matrix for gene latent representations. All matrices for latent representation were generated from the standard normal distribution. The design matrices for $\{D, P,T,W\}$ are $X_{D}^{400\times50}$, $X_{P}^{400\times2}$, $X_{T}^{400\times8}$, and $X_{W}^{250\times16}$, respectively. The noise matrix $\Theta$ (400×1000) was generated from a normal distribution $N(0,\delta)$, and $\delta$ is the variance of the distribution. In our simulations, we considered $\delta$ equal to 0.25, 0.5, 0.75, and 1.

To better mimic the complex missing samples in real settings, we consider the setting based on the distribution of brain structures and phenotypes of samples of 50 donors similar to the ageing, dementia, and TBI data. Among 50 donors, 27 are healthy, and the other 23 are affected by a specific disease. For each donor, at least 4 out of 8 tissues are available. Then, we had 229 samples available, and 171 (42.75%) samples were missing. For comparison, we also conducted simulation studies on the scenario where 42.75% of samples are missing at random. Further, to introduce missing entries in the data matrix of samples available, we randomly drew 5% of the elements from it as missing entries. To evaluate the performance of INSIDER in handling missing, we examined the RMSE between the estimated $\hat{R}$ by INSIDER and $R$ over missing entries and samples, and the result is presented in Table 3. In the table, the baseline RMSE is the root-mean-square difference between $R$ for missing entries or samples and the average of $Z$ over nonmissing entries. Since the baseline RMSE for missing entries and samples are roughly the same, we only show the baseline RMSE for missing samples for illustration.

Table 3. The baseline RMSE and RMSE of the estimated $\hat{R}$ by INSIDER against $R$ on missing entries and samples across different simulation settings

| RMSE  Noise | Baseline | Missing  entries | Random  missing  samples | Missing samples  (Real) |
| --- | --- | --- | --- | --- |
| 0.25 | 4.5308 | 0.0460 | 0.0394 | 0.0401 |
| 0.50 | 4.6308 | 0.0907 | 0.0780 | 0.0803 |
| 0.75 | 4.5998 | 0.1397 | 0.1207 | 0.1206 |
| 1.00 | 4.4998 | 0.1827 | 0.1553 | 0.1581 |

From the table, we see that the RMSE for missing entries and samples increases slightly with increasing noise levels, and the RMSE for missing entries is slightly higher compared with the RMSE for missing samples. The RMSE for random missing samples and the designed missing samples based on the ageing, dementia, and TBI data is roughly the same. The simulation result suggests that the INSIDER has a good ability to recover underlying true patterns and the ability decreases slightly with increasing noise levels, even though complex missing exists.

We have added the above content at the end of the section “Simulation study” to demonstrate the ability of INSIDER to handle missing data.

2. Additionally, as one reviewer highlighted, the process for determining the rank K in the latent space is vague. A more clear, justifiable, and efficient method for the choice is needed.

We thank the editor for the suggestion. To improve the clarity and justifiability of our method in selecting $K$ and also demonstrate its efficiency, we provided the following evidence.

In practice, we first randomly draw 10% of elements without replacement from the data matrix as testset and then treat the kept data as trainset. Only the trainset is used to train INSIDER, and the testset is independent from model training and utilized for model selection. Specifically, we first train INSIDER on the trainset and then examine whether our approximations for the missing entries by the trained model can well approximate the observed values in the testset. This practice is also consistent with the idea of cross-validation, which performs model selection based on model performance on held-out data. In fact, it is also widely used in recommendation systems [6] and matrix completion [7].

To evaluate the performance of INSIDER with different choices of $K$, the root mean squared error (RMSE) on the testset is calculated, which measures the averaged squared difference between the approximations from INSIDER and the true values, so a lower RMSE on the testset corresponds to a better model. In our applications, $K$ was chosen from a sequence from 10 to 30 with step size 2. The range of $K$ is enough in general and can be broadened to consider more complex models. For each try of $K$ from the sequence, we run INSIDER for 30 iterations with hyperparameters $\lambda=0.1$ and $\alpha=0$ and report RMSE on the testset. Then, we select the rank $K$ corresponding to the lowest RMSE on the testset. For demonstration, we applied the above procedure with the BrainSpan dataset and showed the RMSE on the trainset and testset for the rank $K$ chosen from 3 to 50 with step size 2, presented in the figure below.

Figure 1. RMSE on the trainset and testset for different choices of $K$ in application to the BrainSpan data

The figure shows that the RMSE on the trainset decreases with increasing rank $K$, but the RMSE on the testset decreases at the beginning and increases after the complexity of INSIDER is higher than needed. In practice, we choose the rank $K$ corresponding to the lowest RMSE on the testset.

Figure 2. RMSE on the trainset and testset for different choices of $K$ after 30 and 50 iterations in application to the BrainSpan data

Also, we examined the effect of different numbers of iterations (e.g., 30, 50) on model selection. We found that 30 iterations are sufficient to give consistent selection results, compared with more iterations (50 iterations). For demonstration, we show RMSE on the trainset and testset with 30 and 50 iterations for the rank $K$ chosen from 11 to 50 with step size 2, and the results are presented in the figure and table below.

Table 4. RMSE on the trainset and testset for different choices of $K$ after 30 and 50 iterations in application to the BrainSpan data

| Rank *K* | Trainset  (30 iterations) | | Testset  (30 iterations) | Trainset  (50 iterations) | Testset  (50 iterations) |
| --- | --- | --- | --- | --- | --- |
| 3 | 0.40209 | 0.40450 | | 0.40209 | 0.40450 |
| 5 | 0.37800 | 0.38169 | | 0.37800 | 0.38169 |
| 7 | 0.36429 | 0.36994 | | 0.36429 | 0.36994 |
| 9 | 0.35457 | 0.36200 | | 0.35457 | 0.36200 |
| 11 | 0.34810 | 0.35686 | | 0.34810 | 0.35686 |
| 13 | 0.34450 | 0.35479 | | 0.34450 | 0.35479 |
| 15 | 0.34182 | 0.35330 | | 0.34182 | 0.35330 |
| 17 | 0.33992 | 0.35273 | | 0.33992 | 0.35273 |
| 19 | 0.33842 | 0.35261 | | 0.33838 | 0.35256 |
| 21 | 0.33713 | 0.35274 | | 0.33713 | 0.35274 |
| 23 | 0.33628 | 0.35320 | | 0.33628 | 0.35321 |
| 25 | 0.33571 | 0.35397 | | 0.33571 | 0.35397 |
| 27 | 0.33534 | 0.35498 | | 0.33534 | 0.35498 |
| 29 | 0.33503 | 0.35604 | | 0.33503 | 0.35609 |
| 31 | 0.33477 | 0.35723 | | 0.33477 | 0.35725 |
| 33 | 0.33454 | 0.35829 | | 0.33454 | 0.35830 |
| 35 | 0.33435 | 0.35968 | | 0.33434 | 0.35971 |
| 37 | 0.33417 | 0.36135 | | 0.33417 | 0.36159 |
| 39 | 0.33412 | 0.36155 | | 0.33412 | 0.36138 |
| 41 | 0.33412 | 0.36140 | | 0.33412 | 0.36128 |
| 43 | 0.33412 | 0.36149 | | 0.33412 | 0.36131 |
| 45 | 0.33412 | 0.36144 | | 0.33412 | 0.36130 |
| 47 | 0.33412 | 0.36134 | | 0.33412 | 0.36121 |
| 49 | 0.33412 | 0.36144 | | 0.33412 | 0.36127 |

The above figure and table show that choosing $K$ with 30 and 50 iterations gives highly consistent results in terms of RMSE. Previous studies [8], [9] also used a small number of iterations (e.g., 20 iterations) to select the rank $K$ for methods based on matrix factorization. Note that our method for selecting the rank $K$ is quite computationally efficient since we set $\alpha=0$ when we select $K$, and the update for all parameters can be computed in a closed form.

We revised the corresponding content in the section “Initialization, hyperparameter tuning and the stopping criterion” in the manuscript to improve the clarity and justifiability of our method and show its efficiency.

3. Regarding the analysis of real data, the comparison to standard and frequently used methods like PCA or GLM-based approaches is inadequate. While some discussions are present, more detailed comparative analyses are required.

We thank the editor for the insightful comment. Indeed, comparative analysis in real data is an effective and direct way to demonstrate the superiority of methods if the methods can be directly compared. Because the research question that PCA and GLM-based methods address is different from that that in INISDER, direct comparisons between INSIDER and methods like PCA or GLM-based approaches may not be applicable. We apologize for the confusion due to our unclear writing on this part. The reasons are listed below. Further, we revised the corresponding content to clarify the confusion.

Here we would like to demonstrate the difference between INSIDER and PCA/factor analysis, as well as GLM-based approaches in depth. For illustration, assume that $Z^{N\times M}$ is the expression of $M$ genes for $N$ samples and that $X^{N\times p}$ is a covariate matrix for $p$ categorical variables, such as donor, tissue, phenotype.

In PCA/factor analysis, the variation of $Z^{N\times M}$ is modeled by ${Z^{N\times M}=H}^{N\times K}V^{K\times M}+\Theta$, where $\Theta$ is the matrix for noise. Both $H^{N\times K}$ and $V^{K\times M}$ are unobserved. After performing PCA/factor analysis, we have latent embeddings $H^{N\times K}$ for $N$ samples. For the GLM-based approaches for differentially expressed analysis, the variation of $Z^{N\times M}$ is modeled by $X^{N\times p}\beta^{p\times M}$. Here $\beta^{p\times M}$ is the coefficient matrix of $p$ covariates for $M$ genes and is used for differentially expressed analysis for the categorical covariates like phenotypes. For INSIDER, the variation of $Z^{N\times M}$ is approximated by $Z^{N\times M} \approx\left( X_{D}D+X_{P}P+X_{T}T+X_{W}W \right)V$. Here $X_{D},X_{P},X_{T},X_{W}$ are the dummy indicator matrices for donors, tissues, phenotypes, and the interaction between phenotypes and tissues, rows of $V$ are metagenes, which capture genes that co-express or work together to induce biological functions, and $D,P,T,W$ are the levels of expression of metagenes (i.e., sets of biological pathways) for different covariates. INSIDER also considers the sparsity and grouping effects of genes by introducing elastic net penalty.

We see that INSIDER separately models the variation from different known sources (e.g., donors, tissues, phenotypes, and their interactions) to capture the variation of $Z^{N\times M}$, represented by the terms $X_{D}DV$, $X_{P}PV$, $X_{T}TV$, and $X_{W}WV$. However, PCA/factor analysis ignores the source of variation in $Z^{N\times M}$, as it computes $H^{N\times K}$ for samples without further separating the source of variation with respect to known covariates. Thus, a direct comparison between INSIDER and PCA/factor analysis in real data analysis is not applicable. The matrices $D,P,T,W$ in INSIDER reveal the effect of the covariates on the metagenes (i.e., biological pathways), which PCA/factor analysis cannot provide.

As mentioned previously, GLM-based approaches are usually used for differentially expressed analysis in RNA-Seq studies. They compute the coefficient matrix $\beta^{p\times M}$ for $p$ covariates across $M$ genes. That is, $\beta$ measures the relationship between covariates and genes. However, INSIDER is a statistical approach for exploring the latent structure underlying $Z^{N\times M}$ with respect to known sources of variation. In INSIDER, the variation in $Z$ is modeled by $\left( X_{D}D+X_{P}P+X_{T}T+X_{W}W \right)V$. Here $X_{D},X_{P},X_{T},X_{W}$ are covariates. Rows of $V$ are metagenes and capture genes that co-express or work together to induce biological functions, so each row of $V$ corresponds a set of biological pathways. $D,P,T,W$ are the levels of expression of metagenes (i.e., sets of pathways) for different covariates. Therefore, INSIDER explores the effects of covariates on these metagenes (i.e., pathways) encoded by $V$. We see that INSIDER and GLM-based methods explore different levels of biological information in RNA-seq data. Therefore, a direct comparison between INSIDER and GLM-based methods in real data is also inapplicable.

We added the above content to the section III in Supplementary Content to demonstrate the difference between INSIDER and PCA, as well as GLM-based approaches in depth.

4. Lastly, showing additional simulation results in cases of model misspecification would be informative.

We thank the editor for the constructive suggestion. To show the performance of INSIDER in case of model misspecification, we first included simulation studies of INSIDER under the situation where the simulation data is generated according to SDA [10], a tensor decomposition method. Specifically, we consider a tensor of gene expression $Z$ of dimensions 50×5×200, which is generated according to $Z=T+\Theta$, where $T_{nlg}=\sum_{c=1}^{K} E_{nc}F_{lc}V_{cg}$. Here the rank of latent space $K$ is set to 5, $E$ and $F$ are matrices of dimensions 50×5 and 5×5, respectively, and $V$ is a 5×250 matrix for gene latent representations. These matrices were generated from the standard normal distribution. The elements of $\Theta$ are generated from $N(0,\sigma)$. In practice, we consider $\sigma$ to be 0.25, 0.5, 0.75, and 1. For the application of INSIDER to the simulated data, we flat the tensor $Z$ by its first two dimensions and transform it into a 250×200 matrix. Then, we introduce two design matrices $X_{E}^{250\times50}$and $X_{F}^{250\times5}$ for the first two dimensions of $Z$, and the two design matrices are considered as covariates for INSIDER. Since SDA only has one hyperparameter, the latent rank $K$ (*num_comps*), and does not allow missing entries, we adopt the following procedure in the simulation study. When running SDA, the parameters *N* and *num_comps* are set to 50 and 5 (which equal to the true values of components in the simulation), respectively, and default values are used for other parameters. For INSIDER, we first draw 10% of the elements of $Z$ without replacement as validation set and consider the left data trainset, select the hyperparameters with which INSIDER trained on the trainset performs the best on the validation set, and run INSIDER with selected hyperparameters on $Z$. Then we compared the performance of INSIDER with and without interactions to SDA in recovering the true pattern $T$ using root mean squared error (RMSE). The results of the simulation studies are presented in Table 3.

TABLE 5 Comparison of performance of INSIDER without or with interaction to that of SDA in recovering $T$ using RMSE when the data is generated according to SDA

| RMSE | noise variance $\delta$ | | | |
| --- | --- | --- | --- | --- |
|  | 0.25 | 0.5 | 0.75 | 1.0 |
| SDA | 0.04 | 0.08 | 0.11 | 0.16 |
| INSIDER without interaction | 1.22 | 1.57 | 2.10 | 1.66 |
| INSIDER with interaction | 0.05 | 0.11 | 0.16 | 0.21 |

From the above table, we find that INSIDER without interaction did not perform well in the case of model misspecification. However, INSIDER with interaction demonstrated a performance similar to SDA, although the simulation data is generated according to the SDA model. This suggests that INSIDER performs well in the case of model specification when data is generated according to SDA.

To further demonstrate the performance of INSIDER in case of model misspecification, we conducted further simulation analysis to examine its performance when the data is generated according to multivariate linear regression models. For illustration, the gene expression matrix $Z$ of dimensions 100×200 is generated by $Z=T+\Theta$, where $T=X_{E}E+X_{F}F$, $X_{E}^{100\times20}$ and $X_{F}^{100\times5}$ are the dummy matrices for two categorical variables, $E^{20\times200},F^{5\times200}$ are matrices for coefficients of the two categorical variables across 200 genes, and $\Theta^{100\times200}$ is the error matrix. In simulation, $E$ and $F$ are generated from $N(0,1)$ and $\Theta$ is generated from $N(0,\sigma)$, where $\sigma$ denotes the noise level. We consider $\sigma$ to be 0.25, 0.5, 0.75, and 1. For INSIDER, we incorporate two categorical variables as covariates without considering their interaction. For linear regression, we fit ridge regression with the two categorical variables as predictors and $Z$ as responses. For a fair comparison, we first draw 10% of the elements of $Z$ without replacement as hold-out set, draw 10% of the elements of the left data without replacement as validation set, and consider other elements of the left data as trainset. To employ INSIDER and Ridge regression, we first train them on the trainset with different hyperparameters and select the parameters with which they perform the best on the validation set in terms of RMSE. Then, we fit them with the chosen hyperparameters on the left data (trainset + validation set) and report the RMSE of the fitted model on $T$ for the testset. The baseline RMSE is the root-mean-square difference between $T$ and the average of $Z$ over the trainset and validation set. The results of the simulation are shown in Table 6.

The table below shows that INSIDER performs similarly to ridge regression in terms of RMSE in recovering $T$. Moreover, compared with the baseline RMSE, INSIDER captures a large proportion of signals. This suggests that INSIDER performs well under the case of model specification when the simulated data is generated according to multivariate linear models.

TABLE 6 Performance of INSIDER in recovering $T$ over the hold-out set using RMSE when the data is generated according to a multivariate linear model

| RMSE | noise variance $\delta$ | | | |
| --- | --- | --- | --- | --- |
|  | 0.25 | 0.5 | 0.75 | 1.0 |
| Baseline | 1.4234 | 1.4046 | 1.4005 | 1.4001 |
| Ridge regression | 0.1496 | 0.2934 | 0.4257 | 0.5426 |
| INSIDER | 0.1493 | 0.2950 | 0.4268 | 0.5458 |

We incorporated the above content into the section “Simulation study” of our manuscript to demonstrate the performance of INSIDER in cases of model misspecification.

References

[1] J. A. Miller *et al*, "Transcriptional landscape of the prenatal human brain," *Nature,* vol. 508, *(7495),* pp. 199-206, 2014.

[2] J. Stiles and T. L. Jernigan, "The basics of brain development," *Neuropsychol. Rev.,* vol. 20, *(4),* pp. 327-348, 2010.

[3] J. Lonsdale *et al*, "The genotype-tissue expression (GTEx) project," *Nat. Genet.,* vol. 45, *(6),* pp. 580-585, 2013.

[4] G. Williams *et al*, "The hypothalamus and the control of energy homeostasis: different circuits, different purposes," *Physiol. Behav.,* vol. 74, *(4-5),* pp. 683-701, 2001.

[5] N. R. Eaton *et al*, "An invariant dimensional liability model of gender differences in mental disorder prevalence: evidence from a national sample." *J. Abnorm. Psychol.,* vol. 121, *(1),* pp. 282, 2012.

[6] Y. Koren, R. Bell and C. Volinsky, "Matrix factorization techniques for recommender systems," *Computer,* vol. 42, *(8),* pp. 30-37, 2009.

[7] T. Hastie *et al*, "Matrix completion and low-rank SVD via fast alternating least squares," *The Journal of Machine Learning Research,* vol. 16, *(1),* pp. 3367-3402, 2015.

[8] K. Qian *et al*, "scINSIGHT for interpreting single-cell gene expression from biologically heterogeneous data," *Genome Biol.,* vol. 23, *(1),* pp. 1-23, 2022.

[9] J. Brunet *et al*, "Metagenes and molecular pattern discovery using matrix factorization," *Proceedings of the National Academy of Sciences,* vol. 101, *(12),* pp. 4164-4169, 2004.

[10] V. Hore *et al*, "Tensor decomposition for multiple-tissue gene expression experiments," *Nat. Genet.,* vol. 48, *(9),* pp. 1094-1100, 2016.
